# Comparative evaluation of mechanical properties of leukocyte rich platelet rich fibrin, advanced-platelet rich fibrin, titanium-platelet rich fibrin, selphyl platelet rich fibrin matrix and merisis platelet rich fibrin matrix

**DOI:** 10.2340/biid.v12.44890

**Published:** 2025-11-26

**Authors:** Sphoorthi Anup Belludi, Neha Pradhan, Bobby Shetty, Anirban Chatterjee, Ruchi Banthia, Shruthi Eshwar, G Kavyashree

**Affiliations:** a Department of Periodontics, K.L.E Society’s Institute of Dental Sciences, Bangalore, Karnataka, India; b Department of Periodontics, Maitri Dental College and Research centre, Durg, Chattisgarh, India; c Department of Periodontics, Modern Dental College and Research centre, Indore, Madhya Pradesh, India; d Department of Public Health Dentistry, K.L.E Society’s Institute of Dental Sciences, Bangalore, Karnataka, India; e Department of Periodontics, Oxford Dental College, Bangalore, Karnataka, India

**Keywords:** Blood concentrates, modulus of elasticity, mechanical properties, tensile strength

## Abstract

**Purpose:**

The interest in mechanical properties of membranes derived from different autologous platelet concentrates (PCs) stems from the need to possess certain qualities to bring about the necessary clinical outcomes as regenerative materials. Despite recent advances leading to procurement of membranes from variety of PCs, there is a dearth in studies comparing and evaluating the mechanical characterisation of these potential membranes. Hence, the present study evaluated the mechanical properties of five different PCs.

**Materials and methods:**

9 mL of intravenous blood was taken and collected at different intervals for procurement of Leukocyte Rich Platelet Rich Fibrin (L-PRF), Advanced-Platelet Rich Fibrin (A-PRF), Titanium-Platelet Rich Fibrin (T-PRF), Selphyl Platelet Rich Fibrin Matrix (Selphyl PRFM), Merisis Platelet Rich Fibrin Matrix (Merisis PRFM). Dynamic Mechanical Analysis technique by surface indentation test using a Triboindenter at 200 μN load was performed to analyse the mechanical properties (hardness, storage modulus, loss modulus, and Tan δ) of the samples.

**Results:**

On comparing the hardness, it was found that A-PRF, Merisis PRFM and Selphyl PRFM demonstrated higher hardness. As for storage modulus, loss modulus and Tan δ, PRFM group (Merisis PRFM and Selphyl PRFM) performed significantly (*p* < 0.01) better than PRF group (L-PRF, A-PRF, T- PRF).

**Conclusion:**

Platelet Rich Fibrin Matrix group membranes demonstrated superior mechanical properties in comparison to PRF group membranes, and therefore seem a preferable choice as barrier membranes as well as for various regeneration purposes.

## Introduction

The major goal of periodontal regeneration is to reiterate the crucial stages of wound healing associated with periodontal development in order to re-establish the lost tissues to their original form and function [1]. Various periodontal regenerative procedures such as barrier membranes, autografts, demineralised freeze-dried bone allografts, bovine-derived xenografts, combinations of membranes and fillers, platelet concentrates (PCs) have been explored for their ability to predictably regenerate the periodontium [2].

Lately PCs have garnered a lot of momentum as platelets within it are a source of active metabolites, growth factors, and proteins. These bind within a developing fibrin mesh or to the extracellular matrix, creating a chemotactic gradient and thereby recruiting stem cells, stimulating cell migration, differentiation, and promoting repair and regeneration. This concept paved the way for the development and clinical application of a plethora of PCs in the field of periodontal regeneration [3]. Based on Melcher’s concept, various commercially available collagen membranes have been used for regeneration of lost periodontal structures in the field of periodontics. However, membrane exposure, risk of cross-infection, rejection of membrane, and high-cost factor have limited its use [4]. This led to the quest for newer materials as membranes to overcome these limitations. Though autologous platelet concentrates (APCs) do not demonstrate the properties of a traditional membrane, they do promote tissue regeneration and act as a biological scaffold. *In vitro* studies suggest that fibrin-based membranes support periosteal and osseous cell proliferation more effectively than collagen membranes, while also resisting forces from infiltrating cells and adjacent tissues. However, their use as guided tissue regenerative materials requires sufficient strength and resilience to protect the blood clot and healing process [5–8]. Amongst the following ideal requirements of barrier membranes such as biocompatibility, space-making, tissue integrity, cell-occlusiveness, mechanical strength, degradability and membrane configuration and design, mechanical characteristics and clinical handling will play an important role in the successful clinical guided bone regeneration outcome [9]. Moreover, strong mechanical characteristics of a scaffold provide a more suitable support for regeneration [10, 11]. Various techniques have been developed to obtain different PCs with a lack of understanding of various PCs particularly those of platelet-rich fibrin matrices (PRFMs). To address this, our previous work highlighted the distinctions between platelet rich fibrin (PRF) and PRFMs in terms of preparation methods, specific characteristics, and regenerative outcomes. Few studies have compared the mechanical properties, particularly those of PRFMs [12–15]. Hence, the present *in vitro* study aimed to evaluate and compare the mechanical properties (hardness, storage modulus, loss modulus, and Tan δ) of various PCs [Leukocyte Rich Platelet Rich Fibrin (L-PRF), Advanced-Platelet Rich Fibrin (A-PRF), Titanium- Platelet Rich Fibrin (T-PRF), Selphyl Platelet Rich Fibrin Matrix (Selphyl PRFM) and Merisis Platelet Rich Fibrin Matrix (Merisis PRFM)].

## Materials and methods

### Study participants

Total of 25 participants were recruited from Department of Periodontics, KLE Society’s Institute of Dental Sciences, Bangalore. Blood samples were obtained from these healthy donors aged between 20 and 40 years after a routine haemogram. The inclusion criteria of the study included systemically and periodontally healthy female subjects. The exclusion criteria included participants taking any drug known to affect the number and function of platelets, abnormal platelet count, immunologic diseases, current smokers, and hematologic disorders [16–20].

### Ethical considerations

All enrolled participants provided written informed consent for enrolment and publishing of the acquired data. Ethical approval was obtained from the institutional ethics committee (KIDS/IEC/NOV-19/37) The study was conducted in accordance with the Helsinki Declaration as revised in 2013.

### Sample size estimation

*A priori* analysis using GPower software v. 3.1.9.4 (Statistical Package for Social Sciences [SPSS] for Windows Version 22.0 Released 2013. Armonk, NY: IBM Corp) estimated a minimum of 5 samples per group, totalling 25, as required to study the characterisation among the groups, assuming a type 1 error rate of 5 and 80% power, assuming a large effect size among the groups. Also, apart from analysing the PCs individually and for the ease of interpreting the data, the five PCs were divided into two groups: a PRF group (L-PRF, A-PRF, T-PRF) and a PRFM group (Selphyl PRFM and Merisis PRFM).

### Preparation of platelet concentrates

Adopting a standard phlebotomy procedure, venous blood was drawn from the antecubital vein for the procurement and preparation of all five types of PCs. The centrifuge machine (Remi REMI-8C, REMI, India) was utilised and either the relative centrifugation force (RCF/g-force) or revolutions per minute (RPM) was calculated with the rotor radius of 11.7 cm of the centrifuge based on the standard formula [21] depending on the standard protocols provided for the different PCs evaluated in the present study. The time taken for withdrawal of blood, transfer of blood to the centrifuge, and to compress the sample was standardised [22].

### Platelet rich fibrin group of platelet concentrates

9 mL of intravenous blood was collected by venipuncture of antecubital vein into sterile glass tubes for the procurement of L-PRF and A-PRF respectively and titanium test tube for the procurement of T-PRF, without any anticoagulants. The tube was immediately placed in a centrifuge machine and spun according to the protocol (L-PRF 3,000 rpm for 10 mins; Advance-PRF 1,500 rpm for 14 mins; Titanium-PRF 3,000 rpm for 10 mins) to separate the blood into supernatant plasma and platelet suspension [21, 23].

### Preparation of platelet rich fibrin matrix group of platelet concentrates

For the procurement of Merisis PRFM, 9 mL of intravenous blood was drawn into Merisis tube (Diponed Biointelligence LLP, Bengaluru, India) and centrifuged at 3,400 rpm for 5 min. For the fabrication of Selphyl-PRFM (Aesthetic factors, Wayne, New Jersey, USA), 9 mL of venous blood was taken and collected in a tube which contained trisodium citrate and patented thixotropic separation gel. The tube was then placed in a centrifuge and spun at 1,100 g for 6 min. The supernate in the collection tube was then transferred to a second test tube which contained calcium chloride with the help of a transferring device and was spun at 1,450 g for 15 min.

### Sample preparation for the assessment of mechanical properties

**Figure 1:**
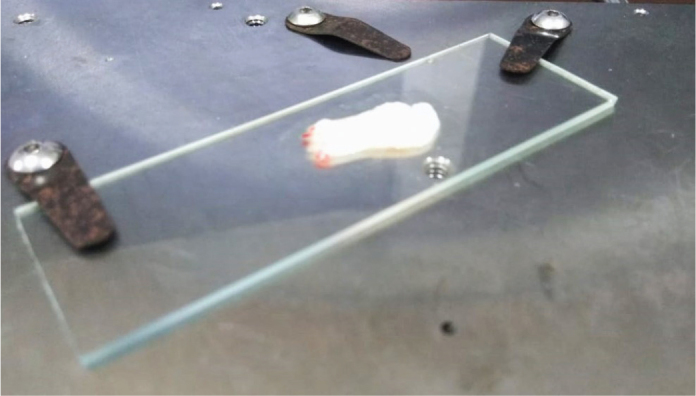
Sample prepared and clamped for assessment in a triboindenter.

Prior to being subjected to mechanical testing, the samples were spread over a glass microscope slide and kept in refrigerator for around 16 hrs to devoid it of saline water. Then the glass slide on the sample stage was clamped using clips (Figure 1).

### Assessment of mechanical properties

The Dynamic Mechanical Analysis (DMA) technique was used to evaluate the mechanical properties of the samples. Surface indentation test with T1 950 Triboindenter (T1 950 Triboindenter, Hysitron Nanotechnology, Minneapolis, US), at 200 μN load was performed to analyse the mechanical properties (hardness, storage modulus, loss modulus, and Tan δ) of PRF and PRFM group of membranes. Indentation load function was defined such that the probe oscillated at a frequency of 210 Hz and at an amplitude of 12 μN during the hold segment. The phase shift between load amplitude and displacement amplitude was measured using Lock-in Amplifier (LIA), and used for the calculation of the mechanical properties. Nanoindentation tests were performed by applying a force to drive an indenter probe (100 μm conical probe) into the sample (loading) surface and then reducing the force to withdraw the probe (unloading) [24].

The storage modulus or young’s modulus is a mechanical property that measures the stiffness of a solid material. It defines the relationship between stress and strain in a material in the linear elasticity region of a uniaxial deformation. While loss modulus describes the material’s viscous response, Tan δ is the ratio of the two dynamic moduli that is storage modulus and loss modulus. In case of elastoplastic samples, storage modulus and loss modulus are comparable to young’s modulus/tensile strength. Measurement of the modulus of elasticity allows separation of a material’s response into its elastic and viscous components under dynamic loading. The **storage modulus (E’)** reflects the stored energy and elastic stiffness, while the **loss modulus (E”)** represents energy dissipated as heat due to viscous effects. Their ratio, **Tan δ (E”/E’)**, quantifies damping. Together, these parameters provide a comprehensive picture of material behaviour, linking stiffness, energy dissipation, and viscoelastic balance.

### Statistical analysis

One-way analysis of variance (ANOVA) test followed by Tukey’s post hoc analysis was used to compare the hardness, storage modulus, loss modulus, and Tan δ results. The level of significance was set at *p* < 0.05.

## Results

### Hardness

**Figure 2:**
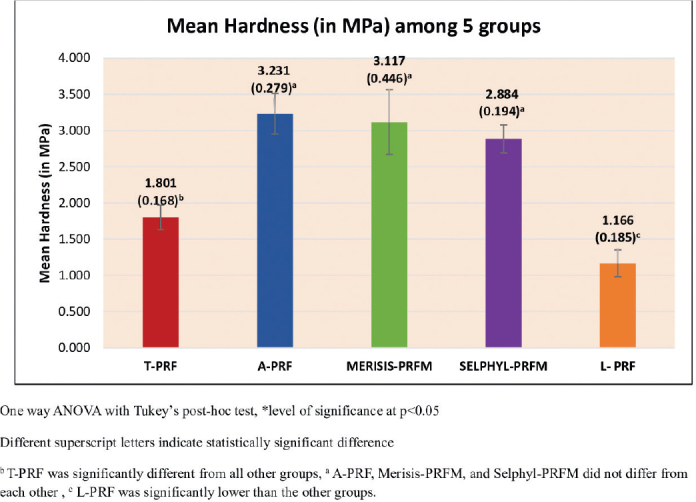
Mean hardness (in MPa) between five groups.

The results of the hardness measurements are presented in Figure 2. Hardness was found to vary with statistical significance (*p* < 0.001). The Tukey’s post hoc tests showed that A-PRF, Merisis-PRFM, and Selphyl-PRFM had statistically similar and significantly higher hardness than T-PRF and L-PRF, and that T-PRF had significantly higher hardness than L-PRF (Table 1).

**Table 1:** Multiple comparison of mean difference in hardness between groups using Tukey’s post hoc test.

(I) Groups	(J) Groups	Mean Diff. (I-J)	95% CI for the Diff.		*p*
			Lower	Upper	
T-PRF	A-PRF	–1.4298	–1.9500	–0.9096	< 0.001*
	MERISIS-PRFM	–1.3154	–1.8356	–0.7952	< 0.001*
	SELPHYL-PRFM	–1.0826	–1.6028	–0.5624	< 0.001*
	L-PRF	0.6354	0.1152	1.1556	0.01*
A-PRF	MERISIS-PRFM	0.1144	-0.4058	0.6346	0.96
	SELPHYL-PRFM	0.3472	-0.1730	0.8674	0.30
	L-PRF	2.0652	1.5450	2.5854	< 0.001*
MERISIS-PRFM	SELPHYL-PRFM	0.2328	-0.2874	0.7530	0.67
	L-PRF	1.9508	1.4306	2.4710	< 0.001*
SELPHYL-PRFM	L-PRF	1.7180	1.1978	2.2382	< 0.001*

CI, confidence interval; T-PRF, Titanium-Platelet Rich Fibrin; A-PRF, Advanced-Platelet Rich Fibrin; L-PRF, Leukocyte Rich Platelet Rich Fibrin; PRFM, Platelet Rich Fibrin Matrix.

### Storage modulus

**Figure 3:**
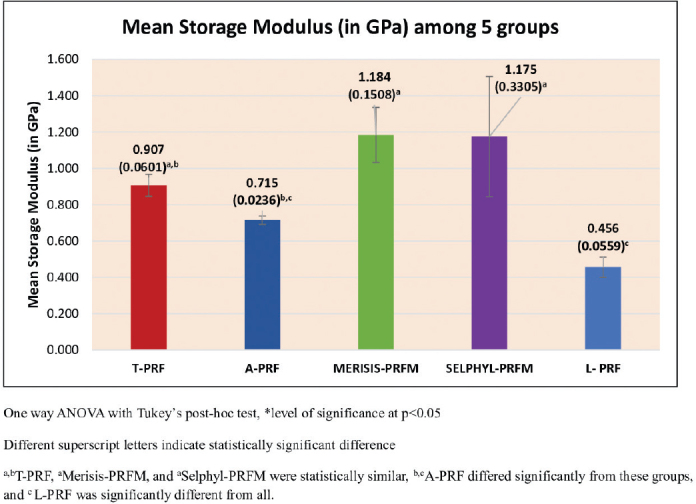
Mean storage Modulus (in GPa) between five groups.

The storage modulus results, presented in Figure 3, were found to vary with statistical significance (*p* < 0.001). The Tukey’s post hoc tests showed that Selphyl-PRFM, Merisis-PRFM, and T-PRF had statistically similar and significantly higher storage modulus than L-PRF. Selphyl-PRFM and Merisis-PRFM also had higher storage modulus than A-PRF. No differences were found between A-PRF and T-PRF, nor between A-PRF and L-PRF (Table 2).

**Table 2:** Multiple comparison of mean difference in storage modulus between groups using Tukey’s post hoc test.

(I) Groups	(J) Groups	Mean Diff. (I-J)	95% CI for the Diff.		*p*
			Lower	Upper	
T-PRF	A-PRF	0.1925	-0.1233	0.5083	0.39
	MERISIS-PRFM	-0.2770	-0.5928	0.0389	0.10
	SELPHYL-PRFM	-0.2678	-0.5836	0.0481	0.12
	L-PRF	0.4514	0.1356	0.7673	0.003*
A-PRF	MERISIS-PRFM	-0.4695	-0.7853	-0.1536	0.002*
	SELPHYL-PRFM	-0.4603	-0.7761	-0.1444	0.002*
	L-PRF	0.2589	-0.0569	0.5748	0.14
MERISIS-PRFM	SELPHYL-PRFM	0.0092	-0.3067	0.3250	1.00
	L-PRF	0.7284	0.4125	1.0442	< 0.001*
SELPHYL-PRFM	L-PRF	0.7192	0.4034	1.0350	< 0.001*

* – Statistically Significant

CI, confidence interval; T-PRF, Titanium-Platelet Rich Fibrin; A-PRF, Advanced-Platelet Rich Fibrin; L-PRF, Leukocyte Rich Platelet Rich Fibrin; PRFM, Platelet Rich Fibrin Matrix.

### Loss modulus

**Figure 4:**
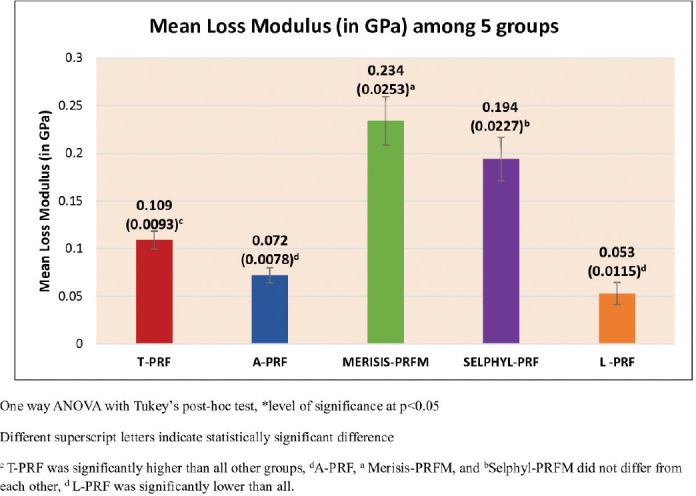
Mean loss modulus (in GPa) between five groups.

The loss modulus results are presented in Figure 4 and were found to vary with statistical significance (*p* < 0.001). The Tukey’s post hoc tests showed that Merisis-PRFM had the highest loss modulus followed by Selphyl-PRFM, T-PRF, A-PRF, and finally L-PRF. All differences were statistically significant except that between A-PRF and L-PRF (Table 3).

**Table 3:** Multiple comparison of mean difference in loss modulus between groups using Tukey’s post hoc test.

(I) Groups	(J) Groups	Mean Diff. (I-J)	95% CI for the Diff.		*p*
			Lower	Upper	
T-PRF	A-PRF	0.0376	0.0056	0.0697	0.02*
	MERISIS-PRFM	-0.1248	-0.1569	-0.0927	< 0.001*
	SELPHYL-PRFM	-0.0849	-0.1170	-0.0528	< 0.001*
	L-PRF	0.0561	0.0240	0.0882	< 0.001*
A-PRF	MERISIS-PRFM	-0.1625	-0.1945	-0.1304	< 0.001*
	SELPHYL-PRFM	-0.1225	-0.1546	-0.0904	< 0.001*
	L-PRF	0.0185	-0.0136	0.0505	0.44
MERISIS-PRFM	SELPHYL-PRFM	0.0399	0.0079	0.0720	0.01*
	L-PRF	0.1809	0.1488	0.2130	< 0.001*
SELPHYL-PRFM	L-PRF	0.1410	0.1089	0.1731	< 0.001*

* – Statistically Significant

CI, confidence interval; T-PRF, Titanium-Platelet Rich Fibrin; A-PRF, Advanced-Platelet Rich Fibrin; L-PRF, Leukocyte Rich Platelet Rich Fibrin; PRFM, Platelet Rich Fibrin Matrix.

### Tan δ

**Figure 5:**
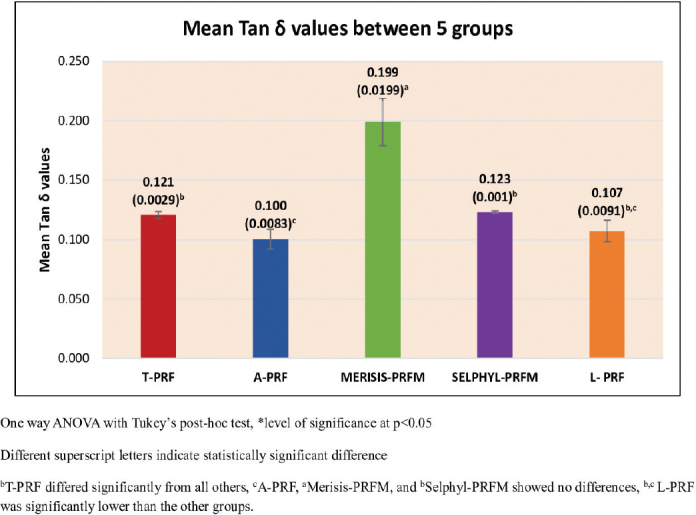
Mean Tan δ values between five groups.

The Tan δ results are presented in Figure 5 and were found to vary with statistical significance (*p* < 0.001). The Tukey’s post hoc tests showed that Merisis-PRFM had significantly higher Tan δ than all other PCs, and that A-PRF had significantly lower Tan δ than all other PCs except L-PRF (Table 4).

**Table 4:** Multiple comparison of mean difference in Tan δ values between groups using Tukey’s post hoc test.

(I) Groups	(J) Groups	Mean Diff. (I-J)	95% CI for the Diff.		*p*
			Lower	Upper	
T-PRF	A-PRF	0.0202	0.0003	0.0402	0.04*
	MERISIS-PRFM	-0.0784	-0.0984	-0.0584	< 0.001*
	SELPHYL-PRFM	-0.0024	-0.0224	0.0176	1.00
	L-PRF	0.0134	-0.0066	0.0334	0.30
A-PRF	MERISIS-PRFM	-0.0986	-0.1186	-0.0787	< 0.001*
	SELPHYL-PRFM	-0.0226	-0.0426	-0.0027	0.02*
	L-PRF	-0.0068	-0.0268	0.0132	0.84
MERISIS-PRFM	SELPHYL-PRFM	0.0760	0.0560	0.0960	< 0.001*
	L-PRF	0.0918	0.0718	0.1118	< 0.001*
SELPHYL-PRFM	L-PRF	0.0158	-0.0042	0.0358	0.17

* – Statistically Significant

CI, confidence interval; T-PRF, Titanium-Platelet Rich Fibrin; A-PRF, Advanced-Platelet Rich Fibrin; L-PRF, Leukocyte Rich Platelet Rich Fibrin; PRFM, Platelet Rich Fibrin Matrix.

Measurement and estimation of hardness, storage modulus, loss of modulus, and Tan δ under load was plotted with overall five readings from different locations for each membrane. The readings were noted and the mean value was computed (Figure S1–S5).

## Discussion

This study aimed to compare the mechanical properties of five PCs – T-PRF, A-PRF, L-PRF, Merisis-PRFM, and Selphyl-PRFM – using DMA. Four properties were evaluated: hardness, storage modulus, loss modulus, and Tan δ. The results highlight distinct differences between PRF and PRFM membranes, largely attributable to their fibrin architecture and preparation protocols.

Hardness: A-PRF demonstrated the greatest hardness and resistance among the three PRfs. This finding is consistent with previous reports and is likely due to the low-speed centrifugation concept (LSCC), which enriches the matrix with leukocytes and platelets [25, 26–28]. Similar outcomes were noted by Pascoal et al. [11, 25], who found A-PRF more resistant than L-PRF. In contrast, Sam et al. [24] observed limited rigidity in PRF membranes, which may restrict their application in guided tissue regeneration (GTR). Our results confirm that A-PRF possesses superior strength, but rigidity limitations remain for other PRF types.

Storage modulus: Both PRFM variants (Merisis-PRFM and Selphyl-PRFM) showed significantly higher storage modulus than PRF membranes (T-PRF, A-PRF, and L-PRF). This can be attributed to differences in polymerisation, which influences fibrin density and stiffness [29]. Lucarelli et al. similarly reported enhanced stiffness in PRFM (FIBRINET) attributable to strong coarse, twisted, and bended fibres arranged in large coiled bundles frequently aggregated in long cables. This arrangement emerged due to maximal centrifugal force during PRFM preparation and could contribute to enhanced mechanical properties of this fibrin-based material, specifically the elastic modulus and the elongation at break [30]. On the other hand, the slow and natural polymerisation that occurs during the centrifugation of PRF produces fibrin fibrillae assembled in equilateral junctions which provides a flexible and elastic fibrin network [8, 29]. There are basically two types of assemblies of biochemical architectures of fibrin fibrillae amidst the gelling process. First type is the condensed bilateral or tetra-molecular junctions and second type is the equilateral tri-molecular connected junctions. This explains why L-PRF and A-PRF, despite lower stiffness, conform well to irregular defects. Earlier reports align with our results, with modulus of elasticity values lowest for L-PRF (98 ± 7 MPa), followed by A-PRF (123 ± 7 MPa), and T-PRF (152 ± 7 MPa) [31]. While Aggour et al. [32] found PRFM more pliable than PRF, Khorshidi et al. [33] reported higher toughness and tensile strain in L-PRF, highlighting variability across studies. Selphyl-PRFM achieved the highest modulus, likely due to secondary centrifugation at higher gravitational force, producing a dense fibrin network [34]. Merisis-PRFM, despite eliminating this step, demonstrated comparable values, suggesting optimisation of its protocol.

Loss modulus and Tan δ: Platelet Rich Fibrin Matrix membranes exhibited higher loss modulus and Tan δ compared to PRF membranes. This indicates a greater ability to dissipate energy under stress, reflecting a more viscoelastic nature. As this is the first study to report these properties in PCs, direct comparison to previous work was not possible. However, the findings expand the understanding of viscoelastic performance and may have clinical implications for membrane durability under functional loading.

The strength of this study is the standardised selection of donors (systemically and periodontally healthy, non-smoking females aged 20–40 years), reducing biological variability in blood components [16–20, 22]. Another strength is the use of the T1 950 Triboindenter, enabling nanoscale DMA of viscoelastic nature of soft samples [35]. This approach provides more precise characterisation than macroscopic universal testing machines used in most earlier studies [9, 18, 25–26]. However, comparison with published data remains challenging due to methodological differences.

## Conclusion

These findings demonstrate that PRFM membranes (Selphyl PRFM and Merisis PRFM) possess superior stiffness and viscoelastic properties (storage modulus, loss modulus, and Tan δ), while A-PRF shows the highest hardness among PRFs. Platelet Rich Fibrin membranes (L-PRF, A-PRF, T-PRF), despite lower mechanical strength, remain advantageous for adaptability to defect morphology. Hence, on the basis of these properties it can be concluded that PRFM membranes are potentially promising as barrier membranes as well as for various repair and regeneration therapies in periodontal and implant defects. Understanding these differences is essential for selecting the appropriate PC type in periodontal regenerative procedures.

## Supplementary Material


