# Supplementary material for: Comparative evaluation of mechanical properties of leukocyte rich platelet rich fibrin, advanced-platelet rich fibrin, titanium-platelet rich fibrin, selphyl platelet rich fibrin matrix and merisis platelet rich fibrin matrix
Source: Biomater Investig Dent. 2025 Nov 26;12:44890. doi: 10.2340/biid.v12.44890 (PMC12661581; doi:10.2340/biid.v12.44890)

Supplementary material has been published as submitted. It has not been copyedited or typeset by Biomaterial Investigations in Dentistry.

Figure S1: Load Vs Displacement Curve

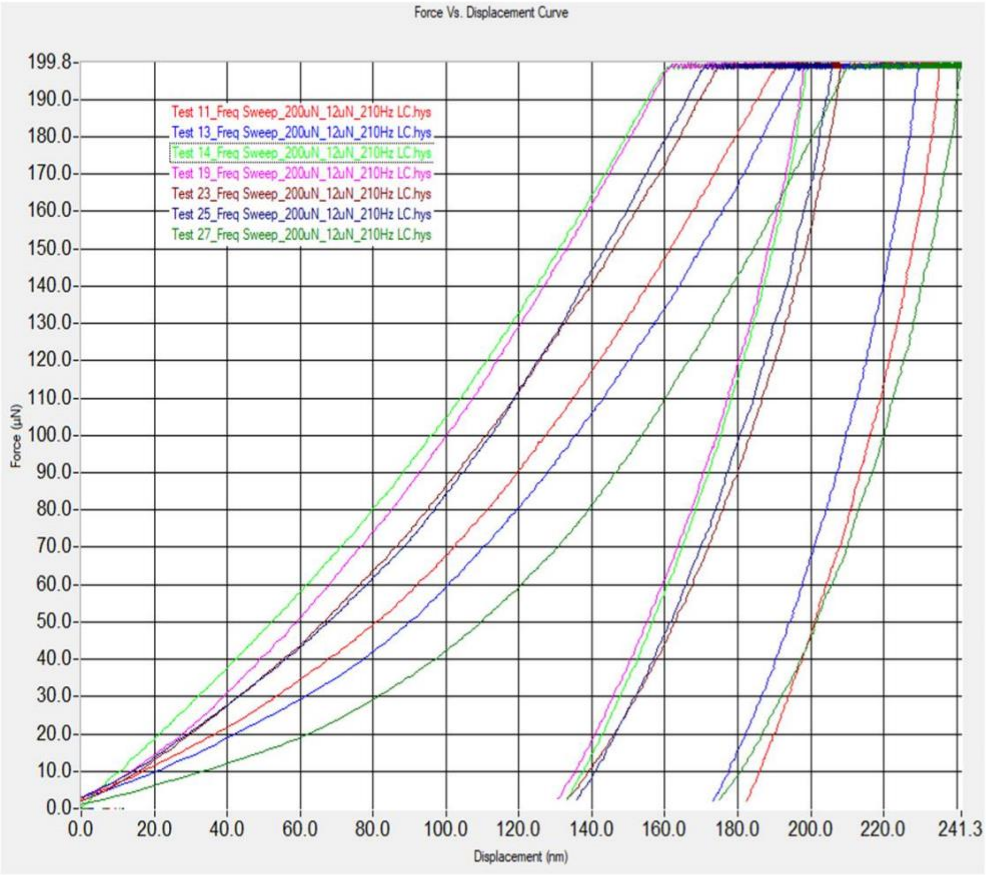

Figure S2: Hardness Vs Frequency Plot

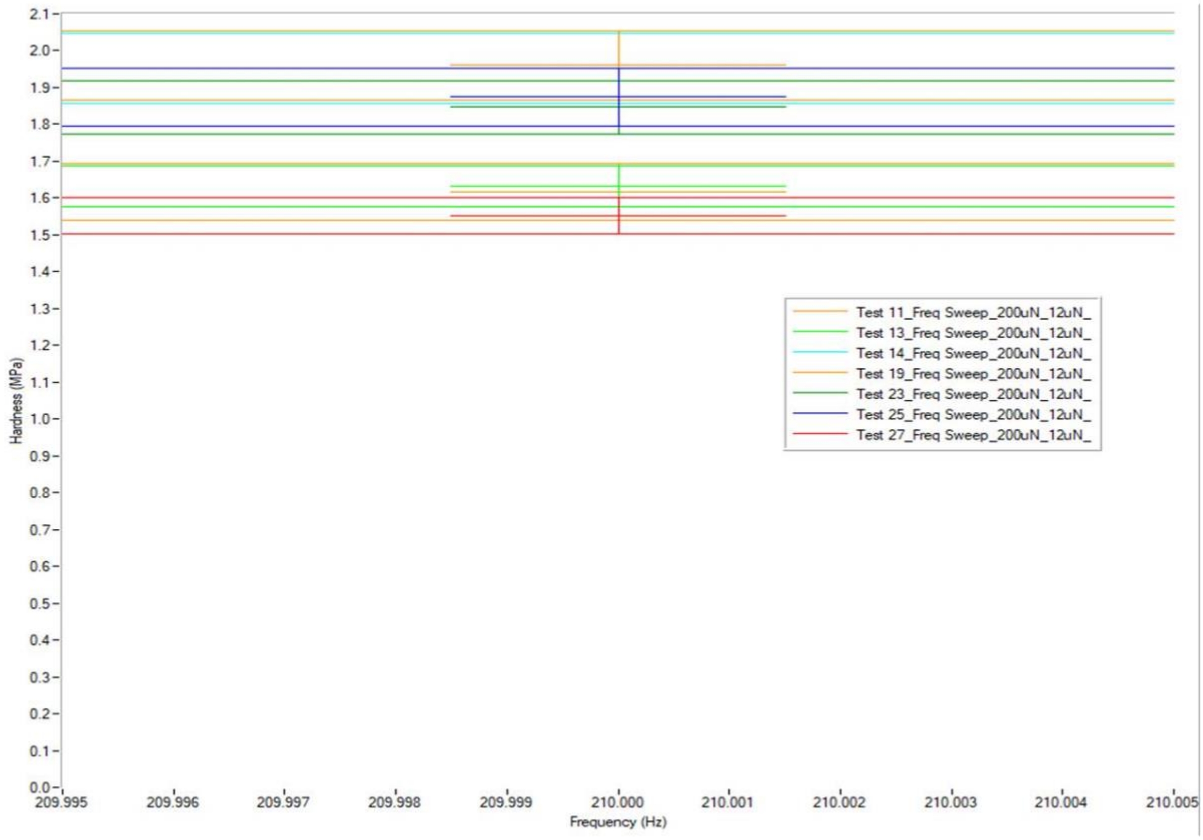

Figure S3: Storage Modulus Vs Frequency Plot

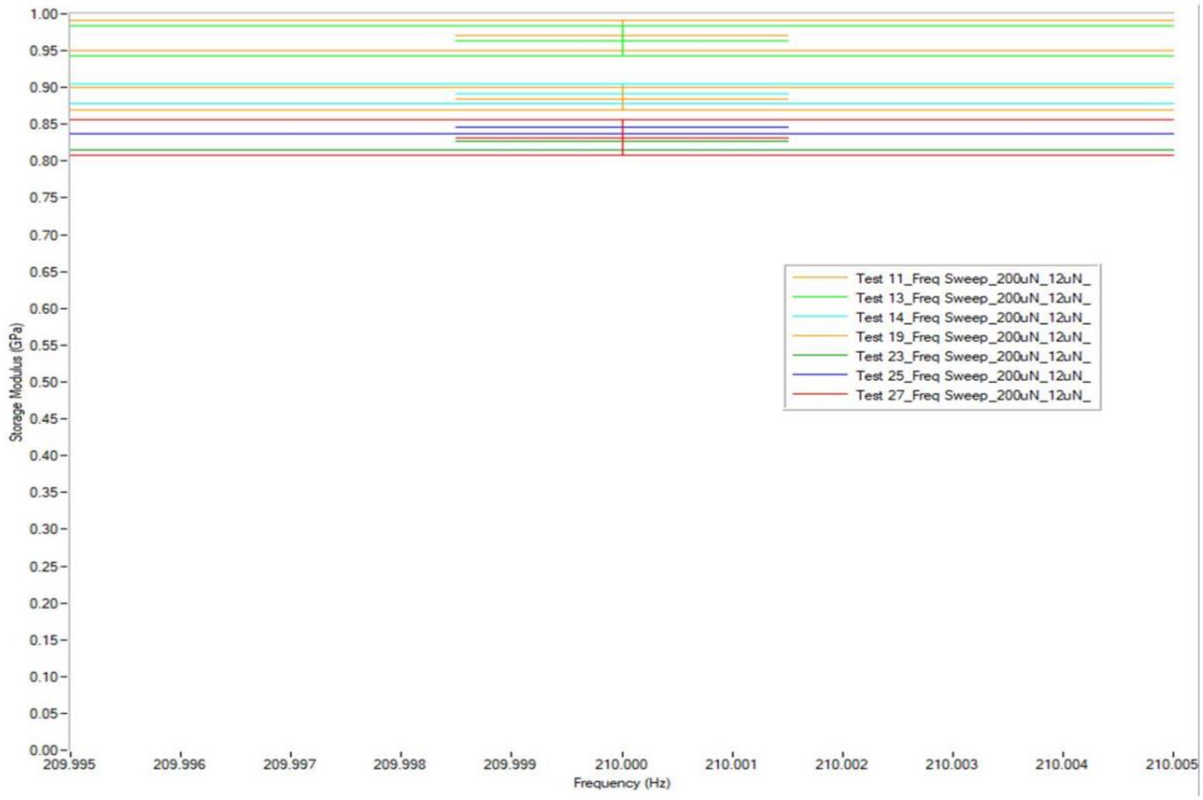

Figure S4: Loss Modulus Vs. Frequency Plot

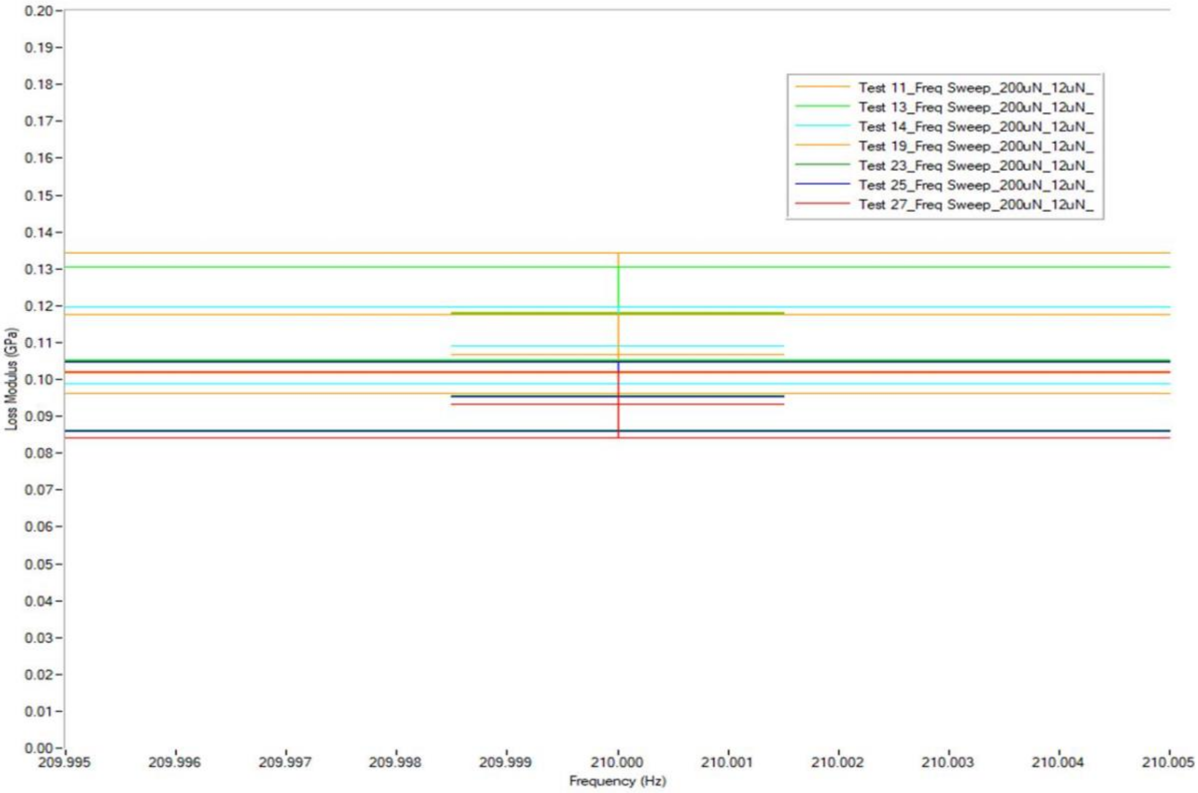

Figure S5: Tan δ Vs Frequency Plot

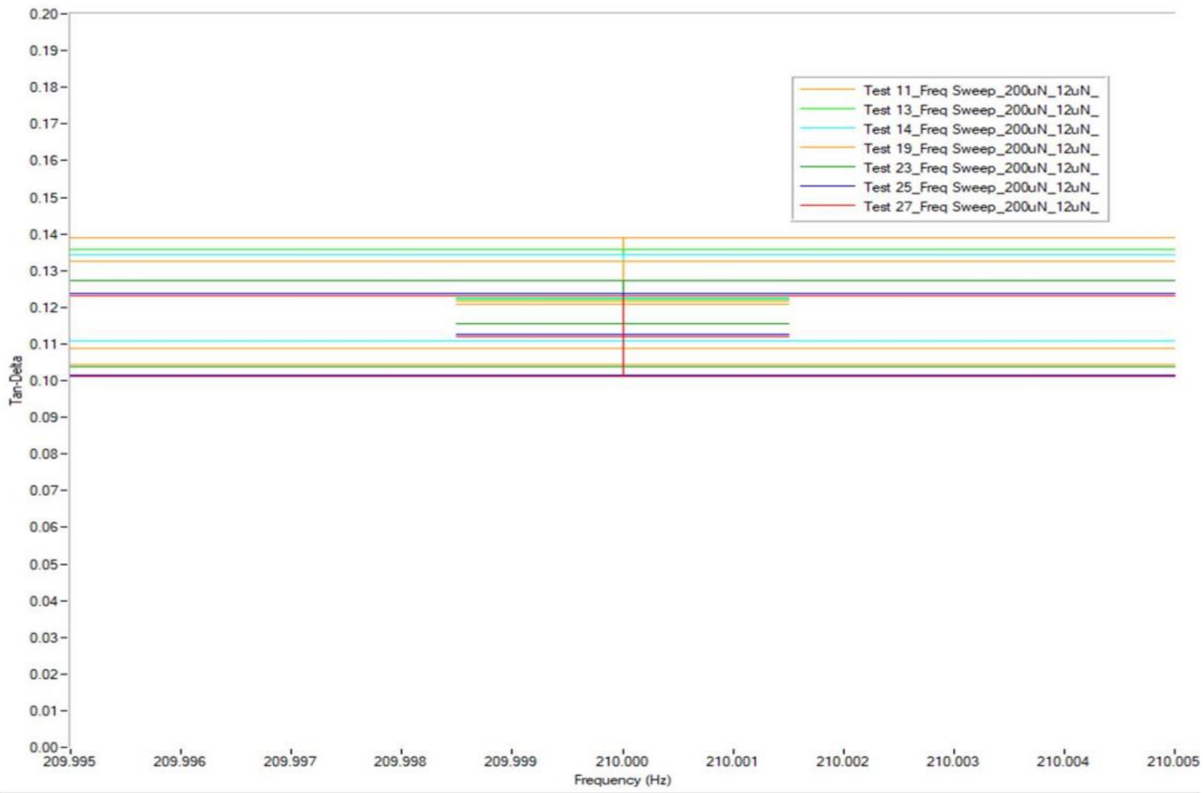

Supplement: Supplementary file 1 [file BIiD-12-44890-s1.pdf]
